# Portal hypertension as the initial manifestation of POEMS syndrome: a case report

**DOI:** 10.1186/s12878-017-0078-8

**Published:** 2017-05-11

**Authors:** Lina Wu, Yue Li, Fang Yao, Chongmei Lu, Jian Li, Weixun Zhou, Jiaming Qian

**Affiliations:** 1 Department of Gastroenterology, Peking Union Medical College Hospital, Chinese Academy of Medical Sciences and Peking Union Medical College, Beijing, 100730 China; 2 Department of Hematology, Peking Union Medical College Hospital, Chinese Academy of Medical Sciences and Peking Union Medical College, Beijing, 100730 China; 3 Department of Pathology, Peking Union Medical College Hospital, Chinese Academy of Medical Sciences and Peking Union Medical College, Beijing, 100730 China

**Keywords:** Portal hypertension, POEMS syndrome, Castleman disease

## Abstract

**Background:**

Portal hypertension has a broad differential diagnosis. POEMS syndrome is an uncommon cause of it. POEMS syndrome is a rare disease involving multiple organs. In differential diagnosis of portal hypertension, POEMS syndrome should be considered especially when other symptoms such as numbness, organomegaly, endocrine alteration and skin changes also present, as it is highlighted by our case.

**Case presentation:**

We report a 46-year-old Chinese male, a teacher, presenting with portal hypertension. Electromyography revealed peripheral neuropathy. Immunofixation showed monoclonal immunoglobulin A lambda protein. The diagnosis of POEMS syndrome was established. After treatment of lenalidomide combined with dexamethasone over 2 years, the patient achieved a considerable improvement.

**Conclusion:**

This case highlights the manifestation of portal hypertension in POEMS syndrome. Lenalidomide with or without dexamethasone is effective for portal hypertension due to POEMS syndrome, though esophageal and gastric varices seems not reversible so easily.

## Background

POEMS syndrome is a rare kind of paraneoplastic syndrome and belongs to plasma cell disorder. POEMS is the abbreviation of polyradiculoneuropathy, organomegaly, endocrinopathy, monoclonal plasma cell disorder and skin changes, which is first proposed by Bardwick in 1980 [1]. POEMS syndrome initially presenting as portal hypertension is seldom [2, 3]. We reported a rare case of portal hypertension appearing as the first manifestation and aggravating during the course of POEMS syndrome. In differential diagnosis of portal hypertension, POEMS syndrome should be considered especially when other symptoms such as numbness, organomegaly, endocrine alteration and skin changes also present. Since portal hypertension may precede other manifestations of POEMS syndrome by a few years, reevaluation of cryptogenic or rapidly progressive portal hypertension over time is important.

## Case presentation

A 46-year-old Chinese male was referred to our hospital in September 2013 because of watery diarrhea and refractory ascites for 2 years. Since May 2013, he developed edema and numbness of the lower extremities. He had some difficulty in walking on himself. Two months later, he had dyspnea especially when he stayed prostration. His body weight decreased 15 kg from October 2010 to October 2011. Due to progressive ascites, his body weight remained stable though leanness continued since then. He had no hematemesis or melena ever before.

Physical exam was notable for cachexia, hyperpigmentation, moderate ascites, jugular venous distention, right axillary lymphadenopathy and splenomegaly. Neurologic examination revealed bilateral lower extremity numbness. The overall neuropathy limitation scale of his arms is 1, and that of his legs is 2. The remaining systemic examination was normal.

**Fig. 1:**
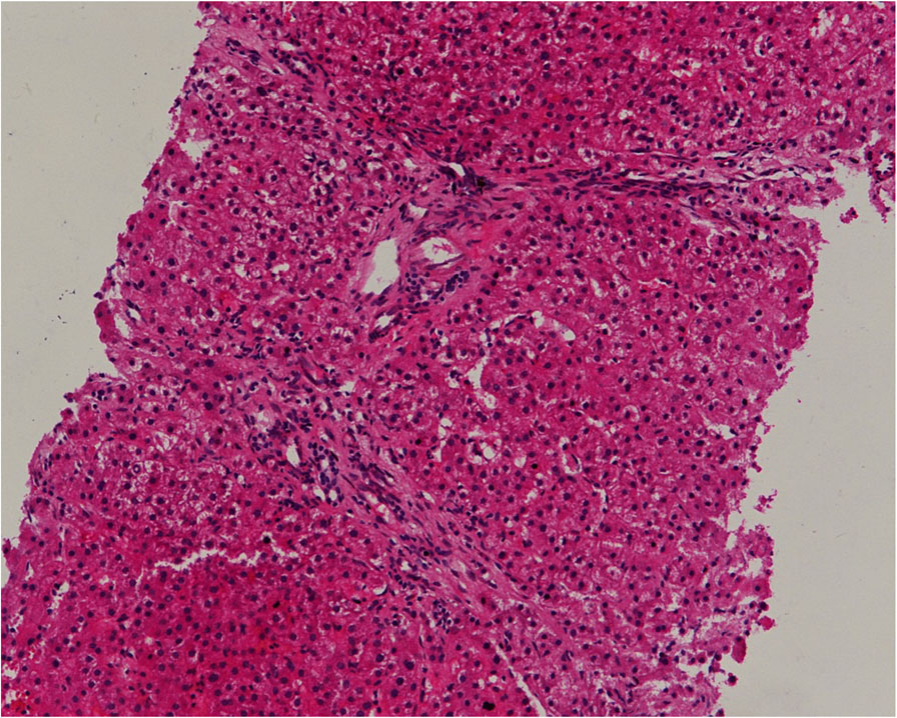
Biopsy of cervical lymph nodes indicated sinus histiocytosis (Hematoxylin-Eosin staining, 200×). The histopathology was compatible with Castleman disease

**Fig. 2:**
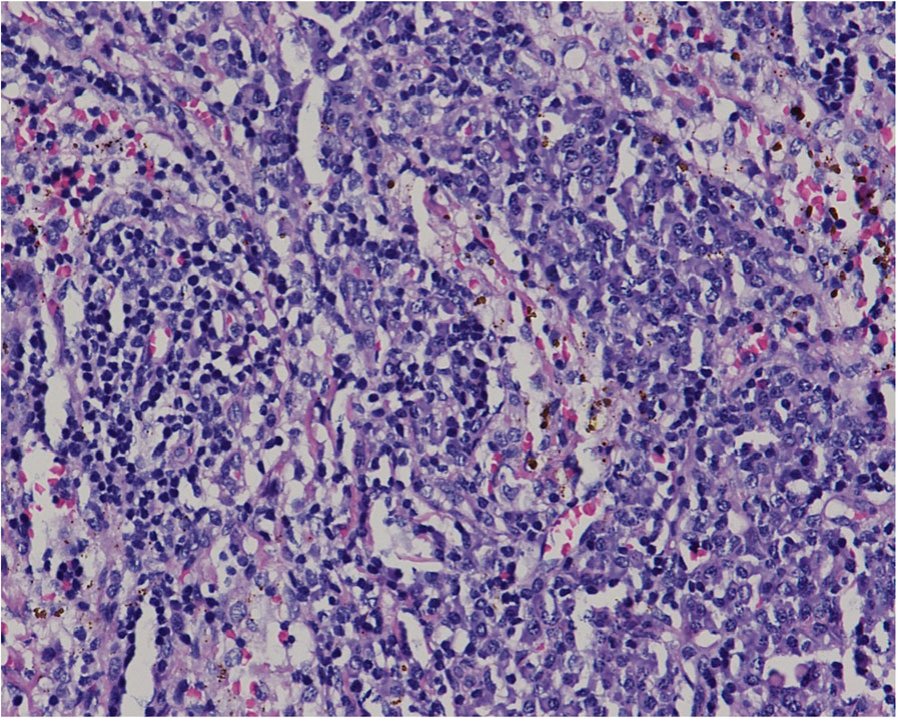
Histopathology of liver biopsy (Hematoxylin-Eosin staining, 100×). The histopathology revealed swelling in part of the hepatocyte, spotty necrosis occasionally and a small quantity of lymphocyte infiltration in portal area, with no evidence of cirrhosis

Complete blood count was within normal range. Liver biochemical tests and electrolytes were normal except mild hypoalbuminemia (23 g/l). Serum creatinine was elevated (123 μmol/L) and 24 h urine total protein was 1.6 g. The screening for antinuclear antibodies and autoimmune hepatitis antibodies were negative. Search for parasites and eggs in stools and viral hepatitis in serum were negative. Monoclonal immunoglobulin A lambda protein was detected in the serum by protein electrophoresis and immunofixation. The serum vascular endothelial growth factor (VEGF) level was significantly elevated (333.1 pg/ml). Endocrine tests confirmed hypothyroidism, hypotestosteronaemia and adrenal insufficiency. Biochemical and cytological analysis of ascitic fluid showed no evidence of infection and malignancy. Serum-ascites albumin gradient (SAAG) is 16 g/l. Ultrasonography showed reduced flow rate of portal vein, phlebectasia of splenic vein, thrombosis of superior mesenteric vein, suggesting portal hypertension. The depth of ascites was 9.3 cm measured by ultrasonography. Echocardiography revealed pulmonary artery pressure of 60 mmHg. Endoscopy revealed severe-grade esophageal and gastric varices and normal colon. Electromyography found peripheral nerve impaired. CT scan documented lymphadenopathy including retroperitoneal lymph nodes fusion, splenomegaly, and dropsy of multiple serous cavity and established collateral circulation of portal system. Neither nodular contour of the liver nor small liver was revealed. Skeletal radiographs and PET/CT revealed lytic lesions with a sclerotic rim on right greater trochanter of femur and densely sclerotic lesions on left ilium. Bone marrow aspirate and biopsy showed plasmocytosis (5%) with apparently normal morphology. Biopsy of his cervical lymph nodes indicated sinus histiocytosis, which was compatible with Castleman disease (Fig. 1). Histopathology of his liver revealed swelling in part of the hepatocyte, spotty necrosis occasionally and a small quantity of lymphocyte infiltration in portal area, which doesn’t support cirrhosis (Fig. 2).

Since the patient presented with polyneuropathy, monoclonal immunoglobulin in immunofixation, Castleman disease, sclerotic bone lesions, serum VEGF elevation,splenomegaly,edema, and endocrinopathy, the diagnosis of POEMS syndrome was established.

After diagnosed as POEMS and Castleman disease, the patient was treated with 12 courses of oral lenalidomide (10 mg/day) on days 1–21 plus oral dexamethasone (20 mg/day) on days 1–4, and 6 courses of oral lenalidomide (10 mg/day) on days 1–21. To decrease the risk of thrombosis by lenalidomide, aspirin at 100 mg/day was administered. Thyroxine and prednisone were used for replacement therapy of endocrine disorder. The patient has been on probiotics and diuretics to control watery diarrhea and ascites. He also received physical therapy and exercise as supportive care.

There was significant improvement after treatment. Pigmentation of his skin lightened. Abdominal distension and splenomegaly relieved. The depth of ascites decreased to 4.5 cm after 12 courses of treatment. Numbness of bilateral lower extremities was in remission. The overall neuropathy limitation scale of his arms is 0, and that of his legs is 0. M-protein was undetectable by serum protein electrophoresis and immunofixation. Echocardiography revealed that pulmonary artery pressure was normal. Esophageal and gastric varices remained no changes and twice nonfatal hematemesis and melena occurred after eight months treated. Endoscopic variceal ligation therapy was scheduled to prevent variceal bleeding. The patient made a good recovery and went to work as usual.

## Discussion

Portal hypertension is rare in POEMS syndrome. Hepatomegaly is the most common hepatic manifestation in POEMS syndrome [4] and 39.6% ~ 54% of patients have ascites [5, 6]. In most cases, characteristics of ascites is non-portal hypertensive and the average SAAG is 6.7 g/l [5]. While in this case, SAAG of ascites fluid is over 11 g/l, accompanied by splenomegaly and esophageal and gastric varices. All evidence confirmed the presence of portal hypertension.

In this case, portal hypertension was probably associated with POEMS syndrome. Since there are no investigations suggested the presence of cirrhosis or other common causes of portal hypertension. Moreover, portal hypertension improved greatly after the systemic treatment of POEMS syndrome. Though the mechanism underlying POEMS syndrome and portal hypertension remains unknown, the association may be not occasional.

The mechanism of portal hypertension occurring in POEMS syndrome is unknown. It was reported that various pathogenic determinants including progressive fibrosis of the portal veins, acquired vascular defect, exposure to several toxins, infections, immunological basis, aberrations in the thrombin-antithrombin complex were related to portal hypertension [7]. Many inflammatory cytokines such as VEGF, Interleukin-1β, Interleukin-6, Interleukin-12 [8], tumor necrosis factor-α [9] are increased in POEMS syndrome. VEGF induces endothelial cells proliferation, migration and microvascular hyperpermeability,and play an important role in angiogenesis [10]. In this case, the VEGF level was significantly elevated in the early stages. Perhaps microvascular hyperpermeability following VEGF overproduction contributes to the onset of portal hypertension. A hepatic circulation defect secondary to POEMS syndrome may contribute to the subsequent portal hypertension.

Melphalan combined with dexamethasone has comparable response rates with autologous peripheral blood stem cell transplantation (PBSCT) [11]. Novel agents such as lenalidomide [12] and bortezomib [13] are suitable for relapsed patients and patients with severe organ dysfunction. But there is no random control trials and multicenter international cooperation.

## Conclusion

This case highlights the manifestation of portal hypertension in POEMS syndrome. Lenalidomide with or without dexamethasone is effective for portal hypertension due to POEMS syndrome, though esophageal and gastric varices seem not reversible so easily.

## Abbreviations

PBSCT: Peripheral blood stem cell transplantation
SAAG: Serum-ascites albumin gradient
VEGF: Vascular endothelial growth factor.
